# Ultrasonic therapies for seizures and drug-resistant epilepsy

**DOI:** 10.3389/fneur.2023.1301956

**Published:** 2023-12-12

**Authors:** Carena Cornelssen, Eli Finlinson, John D. Rolston, Karen S. Wilcox

**Affiliations:** ^1^Department of Biomedical Engineering, University of Utah, Salt Lake City, UT, United States; ^2^Department of Pharmacology and Toxicology, University of Utah, Salt Lake City, UT, United States; ^3^Department of Neurosurgery, Brigham and Women’s Hospital and Harvard Medical School, Boston, MA, United States; ^4^Interdepartmental Program in Neuroscience, University of Utah, Salt Lake City, UT, United States

**Keywords:** focused ultrasound, drug-resistant epilepsy, seizures, therapeutic ultrasound, animal models of epilepsy, clinical epilepsy research, high-intensity focused ultrasound, low-intensity focused ultrasound

## Abstract

Ultrasonic therapy is an increasingly promising approach for the treatment of seizures and drug-resistant epilepsy (DRE). Therapeutic focused ultrasound (FUS) uses thermal or nonthermal energy to either ablate neural tissue or modulate neural activity through high- or low-intensity FUS (HIFU, LIFU), respectively. Both HIFU and LIFU approaches have been investigated for reducing seizure activity in DRE, and additional FUS applications include disrupting the blood–brain barrier in the presence of microbubbles for targeted-drug delivery to the seizure foci. Here, we review the preclinical and clinical studies that have used FUS to treat seizures. Additionally, we review effective FUS parameters and consider limitations and future directions of FUS with respect to the treatment of DRE. While detailed studies to optimize FUS applications are ongoing, FUS has established itself as a potential noninvasive alternative for the treatment of DRE and other neurological disorders.

## 1 Introduction

Epilepsy is a common and costly neurological disorder. Epilepsy is characterized by recurrent spontaneous seizures and affects one out of 26 people worldwide (1). While this is a worldwide disorder, using the Unites States as an example, there are approximately 150,000 new cases of epilepsy per year in the United States, and epilepsy has been estimated to directly and indirectly cost $54 billion a year in the United States if one assumes that 3.4 million people in the US have epilepsy (2–5). Patients with drug-resistant epilepsy (DRE) make up 80% of this cost (2, 6). Therefore, other less invasive therapies for DRE are a critical unmet medical need.

Approximately 30–40% of people with DRE could respond to a more invasive treatment intervention, such as tissue resection surgery or deep brain stimulation, to achieve meaningful reductions in seizures (7–9). Invasive tissue resection surgery to remove the seizure-generating focus is often a successful line of therapy for people with DRE, with at least 50% of people undergoing surgical treatment reaching seizure freedom (10, 11). However, less than 1.5% of people with DRE currently receive this therapy (8, 12). Various barriers prevent or discourage people from undergoing invasive surgery for epilepsy, such as the distribution of information to healthcare providers about the therapy, patient hesitancy, time of recovery, and fear of invasive surgery (3, 11, 13–15). Thus, we need additional, less invasive interventions for people with DRE. The different therapeutic focused ultrasound (FUS) modalities discussed in this review may be that promising intervention. More specifically, high-intensity focused ultrasound (HIFU), discussed in more detail below, can be a direct replacement for tissue resection surgery that eliminates some of the barriers listed previously.

FUS is advantageous over other techniques. Surgical techniques, such as radiofrequency thermocoagulation and laser interstitial thermotherapy involve passing a probe through normal brain parenchyma through a burr hole drilled in the skull and are not truly noninvasive whereas, FUS, is completely noninvasive in humans (13). Additionally, deep brain stimulation and transcranial magnetic stimulation are comparable modalities to LIFU but are either invasive or are not spatially specific and do not target subcortical structures, respectively (16). Therefore, this suggests that FUS may be a leading alternative for noninvasive surgery and neuromodulation therapy, especially when there is a need for focal and subcortical targets.

FUS is a noninvasive brain stimulation approach that uses energy in the form of acoustic waves above the range of human hearing to target a focal area in the brain (15). FUS therapies are discussed in terms of the type of energy or general intensity (power delivered over the tissue area) delivered at the target (17). Currently, the two main modalities of FUS in experimental use are HIFU and low-intensity focused ultrasound (LIFU) (18). HIFU uses thermal energy with high intensity (>200 W/cm^2^), and LIFU uses nonthermal energy with low intensity (<100 W/cm^2^) to affect brain tissue and activity (19).

HIFU holds tremendous potential for people with DRE over invasive surgical options, such as laser interstitial thermal therapy, as it does not involve opening the skull (13). LIFU is advantageous over other noninvasive neurological treatments, such as transcranial magnetic stimulation, because of its greater spatial resolution at depth and can stimulate deep brain areas such as the amygdala and hippocampus (15, 19, 20). Therefore, HIFU and LIFU have increased advantages over other technologies when targeting small and deep brain structures.

## 2 Current studies of HIFU and LIFU demonstrate seizure suppression

### 2.1 HIFU has shown promise for seizure modulation in human studies

HIFU has shown promise for decades in seizure suppression. In the 1960s, researchers used ultrasound lesioning on cats (*n* = 12) induced with seizures by alumina crema to target either the middle suprasylvian gyrus or anterior sigmoid gyrus (21). Ultrasound lesioning resulted in 82% of the animals reaching seizure freedom 12 weeks post-HIFU (21). Today, magnetic resonance imaging (MRI)-guided FUS (MRgFUS) is a HIFU-approved approach by the United States Food and Drug Administration (FDA) for treating essential tremor and Parkinson’s disease (19). The procedure intentionally delivers enough ultrasound to thermally ablate the target region in the thalamus (22). Magnetic resonance guidance (MR-guidance) is used to accurately visualize brain regions while also monitoring the temperature, in real-time, for ablation of the targeted brain region (22). The advantages of MR-guidance make MRgFUS a great noninvasive surgical alternative for patients with DRE. Originally, MRgFUS studies have included studies from non-epileptic human skull with tissue-gel phantoms to demonstrate the possibility of MRgFUS treatment on humans, as limitations of MRgFUS procedures may include increased skull heating (22, 23). Monteith et al. showed that by using the ExAblate Neuro^®^ phased-array system from Insightec, a 30-seconds sonication duration rather than a 10-s sonication duration achieved irreversible lesions in patients with temporal lobe epilepsy (TLE) (23). However, the longer sonication duration of 30 s also generated skull heating (23). Abe et al. were one of the first groups to investigate the treatment for people with DRE with mesial TLE using MRgFUS in humans (24). This group targeted the hippocampus with 12 sonications of 10–12 s duration in one subject to determine safety and efficacy (24). While the procedure did not produce an observable lesion, the patient remained relatively seizure-free for 12 months following the MRgFUS procedure (24). While the patient did report dizziness and headaches during the actual HIFU procedure, no other adverse events were demonstrated (24). However, this study had other limitations, such as a short follow-up period, sub-ablation temperatures below the minimum of 50°C, lack of a discernible lesion, limited sample size, and lack of a control group (24). Another case report of MRgFUS in DRE was recently described for a patient with a hypothalamic hamartoma (25). While this patient was seizure-free one-year post-MRgFUS removal of the hamartoma, the study’s main limitation was that it was a case report with a single patient (25).

The main benefit of MRgFUS for people with DRE, especially TLE, is that now smaller and deeper brain areas, such as the fornix, can be targets, especially when comparing against the current standard of resective surgical treatment, which is a temporal lobectomy (26). MRgFUS has recently been used for DRE to ablate the anterior nucleus of the thalamus in a Phase-1 open-label study in two people with DRE (27). While the primary outcome was safety, a secondary outcome was seizure frequency (27). Safety was based on neuropsychological assessments evaluating language, memory, executive functioning, motor skills, emotional functioning, and quality of life (27). The study had two patients who experienced verbal and attention/working memory issues at the three points of the 12-month follow-up following the procedure; therefore, safety is inconclusive in this study as it could also relate to the lesion’s size or site (27). One of the patients was seizure-free for at least a year, while the other benefitted from a dramatic decrease in seizures from an average of 90–100 seizures per month to around 3–6 seizures per month (27). While open-label studies with small patient numbers have limitations, these preliminary results are encouraging and support the need for additional studies. There are three ongoing clinical trials with MRgFUS for patients with DRE, specifically focal epilepsy. There is one study targeting the epilepsy foci (NCT02804230) in people with DRE that is currently recruiting. There are two studies targeting the anterior nucleus of the thalamus. One of those studies is recruiting (NCT03417297). The second of those studies (NCT05032105) is not yet recruiting but only offers the trial to people with DRE who are comorbid with anxiety. It is exciting that MRgFUS trials are underway for a much-needed patient group, and it will certainly be interesting to see the effects of MRgFUS on varying targets and disease states. These three clinical trials evaluating the effect of HIFU ablation for people with DRE are summarized in Supplementary Table S1, whereas Supplementary Table S2 includes published work on HIFU lesioning in people with DRE.

### 2.2 LIFU has shown promise for seizure modulation in animal studies

LIFU has been used in animal and human studies to affect neural activity and has been shown to suppress electrographic seizure activity (28–37). However, as seen in Supplementary Table S2, which describes the studies in terms of FUS parameters, animal models used, and stimulation targets, most animal studies were conducted in evoked seizure models and not in a chronic disease model of epilepsy (28–32, 34–37). Conducting these studies in an evoked seizure model is the main limiting factor to these studies. There continues to be a need for more studies in animal models of epilepsy to understand the FUS parameters that affect the disease state. In a recent study from 2020, LIFU suppressed seizures in a penicillin-induced nonhuman primate seizure model through stimulation of the prefrontal motor cortex with numerous FUS stimulation parameters (37). The FUS parameters that reduced the number, duration, and frequency of seizures and increased the inter-seizure interval duration for 7 h post-FUS were an ultrasound frequency of 800 kHz, a pulse repetition frequency (PRF) of 500 Hz, a duty cycle of 36%, an intensity of 1 MPa, and a stimulation duration of 15 min (37). These effects were not seen when using a 750 kHz frequency with 5-, 30-, or 60-min FUS duration (37). Limitations in this study included a small sample size of two and using an induced seizure model rather than a chronic disease model of epilepsy (37). Additionally, the lack of sufficient information regarding their sham protocol to understand if the auditory artifact (discussed in the “*Limitations of focused ultrasound for the treatment of epilepsy*” section below), which occurs as a consequence of activating the auditory network via vibrations across the skull from LIFU stimulation, was controlled for properly was another limitation (37). Another recent study using a penicillin-induced nonhuman primate seizure model used a single-element transducer, an ultrasound frequency of 750 kHz, a PRF of 1 kHz, a duty cycle of 30%, an intensity of 0.35 MPa, and a stimulation duration of 30 min (38). A histological study was performed on one nonhuman primate 30 min post-FUS stimulation, and the tissue was found to be intact; therefore, LIFU was deemed to be a safe treatment (38). Additionally, a significant reduction in seizure count and seizure frequency per hour was seen 8 h post-FUS (38). However, this study lacked both a control that rules out the auditory artifact as the potential reason for the effect seen and the use of a chronic disease model (38). Nevertheless, these nonhuman primate studies were important in showing the safety and efficacy of using LIFU for seizure suppression, even though they did not use a chronic disease model of epilepsy.

There has been more research in rodent models of seizures or epilepsy with LIFU than in nonhuman primates. One of the first studies of LIFU stimulation in rodents was in 2011 (32). This study noted a suppression in the number of epileptic bursts and theta band peaks in a rat pentylenetetrazol (PTZ) model of seizures (32). This study used a single-element transducer, a frequency of 690 kHz, a PRF of 100 kHz, and a stimulation duration of 36 min (32). They did not use a control for a potential auditory artifact, and their results cannot be interpreted as only being attributed to a targeted neuromodulation effect (32). Additionally, they did not use a chronic disease model (32). A group in 2015 was one of the first to study LIFU in a chronic mouse model of epilepsy (29). They studied the effects of LIFU on seizures induced by hippocampal infusion of KA and later saw a reduction in spontaneous recurrent seizure activity and improved behavioral measures in the animals that received LIFU (29). This group used a single-element transducer, a frequency of 200 kHz, a PRF of 0.5 kHz, a duty cycle of 50%, and a stimulation duration of 30 s per seizure (29). They noted a decrease in seizures and improved performance in behavioral tasks during the chronic period of epilepsy (29). While this 2015 study used a chronic disease model, they did not use a control for the auditory artifact, and thus, the auditory artifact cannot be ruled out as the reason for the LIFU’s effects (29). A major study in the area of LIFU for DRE using rodents was from a 2020 study, which investigated six different FUS stimulation parameters on seizure suppression (28). This study used a single-element transducer, a frequency of 500 kHz, a PRF of 0.1 kHz, a duty cycle of 0, 3, or 0.8%, and stimulation durations of 0 s, 600 s, or 100 s (28). The higher duty cycle and longer stimulation durations saw a correlation between the safety parameter, Mechanical Index (discussed in the *FUS parameters used and FDA safety guidelines* section below), and spike suppression (28). Additionally, FUS stimulation parameters showed a decreased activation of the mTOR pathway (28). Again, this study did not use a chronic disease model and did not control for the auditory artifact (28). A group recently studied the effect of LIFU on brain connectivity in a kainic acid (KA) intraperitoneal (i.p.) injection rat model (36). Using a single-element transducer, a frequency of 500 kHz, a PRF of 1.5 kHz, and a duty cycle of 50%, the group showed that the brain network connection strength was significantly decreased using measurements of the path length and local and global efficiency among their indicators (36). They also observed that FUS stimulation caused the power in the delta and theta bands to decrease (36). While these are important findings of LIFU on brain connectivity and activity, the study did not include the use of multiple FUS parameters and their findings were in an acute seizure-induced model (36). Additionally, when looking at the characterizations of the pressure field and the intended target, the hippocampus, the ultrasound targeted more than just the hippocampus (36). When studies use rodents, the stimulation areas tend to be larger than the intended target, and this can be hard to discern the impact on just the targeted area. In another study, this group investigated the use of both pulsed and continuous LIFU modes using a single-element transducer, a frequency of 500 kHz, a PRF of 1.5 kHz, and a stimulation duration of 40 min (39). Using an i.p. injection of kainic acid (KA) to induce seizures in rats, this group found that the power in the delta, theta, and alpha bands decreased significantly because of LIFU (39). However, while this decrease was seen after stimulation, there was no significance between the two different pulsing modes, and they did not control for the auditory artifact (39). Power in the delta, theta, and alpha bands decreased during FUS in a model of epilepsy is an interesting finding to begin to discern the direct effect of FUS on brain activity. However, this study was deficient in testing additional FUS parameters and studying the effects in a chronic disease model. More recently, in 2022, research in the intrahippocampal kainate mouse model of TLE, targeting LIFU to the hippocampus that was contralateral to KA injection showed a short-lasting decrease in the occurrence of spikes (40). This decrease in hippocampal spikes suggests that there could be the potential to suppress seizures using a custom fiber photometry coupled focused ultrasound system (40). Besides some clear limitations of controlling for the auditory artifact, stimulating more than the target, studying effects in a seizure model, and needing a stimulation parameter study on a chronic disease model, these studies show clear effects of LIFU stimulation on seizure suppression in rodents.

Aside from rodent studies, human studies have been performed with LIFU stimulation. However, there are very few human studies that have been done with LIFU stimulation in DRE. One of the first studies in 2021 assessed FUS for TLE for safety (33). This team used the BX Pulsar transducer with a frequency of 650 kHz, a PRF of 0.25 or 0.1 kHz, a duty cycle of 50% or 5%, a stimulation duration of 8 × 0.5 s or 2 × 30 s on eight patients (33). Using histological testing, the tissue post-FUS, which was removed post-resection surgery, was not destroyed and, thus, deemed safe for stimulation in humans (33). As this was a study performed on patients receiving tissue resection surgery for the treatment of DRE, there was no long-term follow-up and future work investigating the long-term impact of LIFU on the treatment of seizures is warranted (33). A clinical study, (NCT03860298) published in 2022, assessed the safety of a LIFU device and the effect of FUS on seizures (41). They used the NaviFus™ multi-phased array system to stimulate six patients at the seizure onset zone, with a duty cycle of 30% and a stimulation duration of 30 min, showing various effects on seizures and the frequency of EEG waves (41). In one-third of the patients, a decrease in seizures and a greater decline in power of the theta, alpha, and beta bands over multiple sessions were seen (41). However, one-sixth of the patients saw an increase in seizures, whereas one-third saw an increase in interictal spikes (41). The study was not without its limitations, as the follow-up period was brief, just 72 h, and adverse events included scalp heating and transient naming and memory problems (41). However, these adverse events resolved after a few weeks (41). Following this safety study, this team is performing another clinical trial (NCT04999046) using the NaviFUS™ system, which allows for an in-office treatment with a neuronavigational system (41). They are studying the effects of FUS stimulation on people with DRE over a two-month follow-up period, with outcomes including seizure frequency, anxiety, and depression effects measured by self-report metrics. The limitations here involve using self-report metrics as the main measured outcome in the study. However, nonetheless, this is an important step forward in understanding a potential treatment for DRE. Aside from using a commercial device, another research group has developed their own experimental LIFU setup for stimulating people with DRE (42). In a clinical study (NCT03868293), they used a single-element transducer, a frequency of 548 kHz, a PRF of 0.5 kHz, a duty cycle between 36 and 50%, and a stimulation duration of 140 s per target (42). They have targeted the hippocampus in one patient with TLE and have not experienced adverse events (e.g., scalp heating) (42). The effects on seizure modulation are still being studied and have not yet been published. Skull shape and size can affect targeting and skull heating during FUS and having a small sample size like this study limits the understanding of these potential effects from the setup on a general patient group. This study shows the promise of using non-commercial setups, which can be costly, in a research setting. Additional clinical trials are summarized in Supplementary Table S1 of LIFU for DRE.

As shown, there is a general trend of more studies involving LIFU for DRE than any other FUS therapies for DRE, and there are various effects depending on the FUS stimulation parameters used. Therefore, further studies are needed to investigate the effects of FUS stimulation parameters in the chronic disease state. Clinical trials evaluating the effect of LIFU for DRE are summarized in Supplementary Table S1. Supplementary Table S2 summarizes the published work discussed here and additional works, beyond the scope of the present review, on LIFU stimulation for DRE. While both HIFU and LIFU show promising results in current studies for seizure suppression, much work is needed to determine which FUS parameters result in optimal seizure suppression.

## 3 Targeted-drug delivery with ultrasound as a potential therapy for DRE

HIFU and LIFU are not the only ultrasound therapies for DRE. Using ultrasound to target drug treatments to specific seizure-generating brain areas could limit systemic side effects of antiseizure medications (ASMs). Additionally, this ultrasound therapy can provide new drug therapy options for classes of pharmaceuticals that cannot cross the blood–brain barrier (BBB). Targeted-drug delivery is done primarily in two ways. The first approach disrupts the BBB in precise locations due to targeted LIFU with the use of microbubbles. This microbubble approach allows previously impermeable molecules to pass through the BBB at or near the seizure foci. The second approach uses FUS to target the uncaging of lipophilic agents (e.g., propofol) from nanoparticles without using intravenous microbubbles in precise anatomical locations (43, 44).

BBB opening through the pressure of ultrasound in the presence of microbubbles is currently being explored for clinical application and has shown to transiently open the BBB for 24–72 h (44, 45). Microbubbles, approved by the FDA for use as a contrast agent for diagnostic imaging ultrasound, are gas bubbles less than 5-micron diameter in size (44, 45). Microbubbles are now being investigated for therapeutic purposes by reversibly opening the blood–brain barrier through cavitation from the alternating pressure applied from ultrasound (44, 45). Microbubbles are injected intravenously, and once circulated, LIFU can stimulate the targeted area in the brain with peak pressures between 0.1 and 0.6 MPa, depending on the microbubble sizes, and with frequencies typically around 0.25 MHz (44, 45). Molecules unable to pass through the BBB previously can enter the brain where the BBB is transiently opened at the area where cavitation occurred in the membrane from microbubbles and LIFU stimulation (44). Thus, this targeted-drug delivery approach provides therapy in a localized manner in specific brain regions (44). The research in FUS-mediated BBB opening with microbubbles has shown that it may be a potential application and adapted for humans with DRE as it has been safely used in patients with other neurological disorders targeting the hippocampus and prefrontal cortex (44, 45). However, as discussed thoroughly in the review by Konofagou et al. (44) ultrasound procedures and pressures need to be within the researched parameters that can knowingly avoid unwanted cavitation or damage to blood vessels.

As mentioned above, LIFU can also be paired with a drug carrier such as nanoparticles (43). Nanoparticles have a diameter on the scale of less than 100 nm and have shells made of perfluorocarbon with a gas or liquid core that can cage lipophilic drugs of choice (43, 46, 47). Nanoparticles can be intravenously delivered, and LIFU can transiently and locally target drug delivery directly to a specific brain area (43, 44). However, there are limitations to this method. When intravenously delivered, researchers have found that not all of the drug is delivered locally, and the encapsulations used to deliver the drug across the BBB may be toxic (44). Additionally, this is a costly technique to study (44).

Nonetheless, targeted-drug delivery with FUS and nanoparticles is an important technique to mention as it could provide relief for people with DRE. This technique has been used to disrupt seizure activity in the PTZ-induced seizure rat model (43). This study disrupted seizure activity after delivering propofol-loaded nanoparticles for two 60-s sessions at a maximum peak amplitude of 1.5 MPa using MR-guided LIFU (43). With this same paradigm, mean broadband and theta power declined significantly (43). Furthermore, propofol concentrations showed no increase in the serum level when measured for 10 min post-FUS (43). Blood serum levels that show no increase in the drug concentration post-FUS indicate that with propofol encapsulations and LIFU stimulation, there may be a potential to overcome the method limitation mentioned in the previous paragraph that all of the drug may not be delivered locally (43). Interestingly, a group using a pilocarpine-induced model of epilepsy used LIFU to open the BBB with MR-guidance to deliver quinolinic acid to create lesions in the brain at the hippocampus (48). Even though this is a lesion approach, it is discussed here as they use the targeted-drug technique and LIFU stimulation (48). They reduced the frequency of seizures in mice (*n* = 11) by an average of 21.2%. However, the seizure frequency varied as a function of the areas of neuronal loss, which is summarized in Supplementary Table S2 (48). Key findings showed that bilateral damage to the septal hippocampus increased seizure frequency, while those without bilateral damage to the septal hippocampus and with damage only in the intermediate hippocampus decreased seizure frequency following a 30-day post-FUS period (48). Additionally, an animal which did not have a complete lesion (neuronal loss) showed increased seizure frequency (48). This group used a phased-array system, a 1.5 MHz frequency, a PRF of 0.001 kHz, a duty cycle of 2%, and a stimulation duration of 120 s with multiple sonications (48). The limitation of this study is that even with MR-guidance, there can be incomplete lesions. This same group repeated a similar experiment in the pilocarpine-induced rat model of epilepsy (49). The FUS parameters were similar, apart from using a 650 kHz frequency and a 90-s stimulation duration when targeting the hippocampus (49). Again, this group used quinolinic acid to induce lesions (49). They noticed a general decrease in the number of seizures in the FUS-treated groups, and one-third of these rats did not have convulsive seizures during the 30-day follow-up period (49). While targeted-drug delivery has shown promise in rodent models of epilepsy to modulate seizures, this methodology needs to be studied in humans with DRE, and further study of drugs to encage that could be delivered locally is needed. Targeted-drug delivery with ultrasound is a promising opportunity for people with DRE.

## 4 FUS parameters used and FDA safety guidelines

When designing experimental and clinical studies with FUS, it is important to consider the FUS parameters to be used and to follow safety guidelines to prevent unwanted tissue damage or side effects (50). Unwanted effects, such as cavitation and tissue heating, are supposed to be limited by using the FDA safety parameters; however, other effects, such as undesired behavioral changes, can still occur (51). Interestingly, current safety parameters for therapeutic FUS are based on the FDA guidelines for diagnostic imaging-based ultrasound and are not based on treatment/therapeutic applications for the brain (19, 50, 52). Additionally, diagnostic ultrasound is generally performed at a lower power than therapeutic ultrasound in the brain (53). Furthermore, diagnostic ultrasound involves pulsing sonication for a very brief duration with a frequency greater than or equal to 5 MHz, which varies from the transcranial sub-MHz frequency (53). Thus, optimal stimulation parameters for transcranial applications may be difficult to discern as FDA guidelines use diagnostic ultrasound criteria (19, 50, 52).

The four acoustic factors that are included in the FDA safety guidelines for diagnostic ultrasound are: (1) spatial-peak temporal-average intensity (I_SPTA_); (2) spatial-peak pulse-average intensity (I_SPPA_); (3) mechanical index (MI); and (4) thermal index (TI) (51). I_SPTA_ and I_SPPA_ are in units of W/cm^2^ (19). The safety parameters detail the maximum allowed intensity delivered to the tissue (19). The upper limit set forth by the FDA for these sonication parameters, when applicable, is displayed in Table 1 (19). However, these maximum limits for the parameters are set for the ultrasound focus (the convergence of ultrasound beams at the brain target) (50, 53). These limits can be less than what is needed for neuromodulation FUS, even though it is stimulating at a higher power but at a lower frequency than diagnostic ultrasound (50, 53). These parameters are currently being researched for LIFU to determine efficacious treatment at the maximum intensity levels before unwanted side effects occur in LIFU stimulation (19, 52, 54).

**Table 1:** Safety and common parameters of FUS.

**Definitions**	**FUS parameter definitions**	**Commonly used terms**	**FDA limits**	**HIFU or LIFU**
Spatial-peak temporal-average intensity (I_SPTA_)	Average intensity of the FUS waveform over sonication duration	Not applicable (NA)	720 mW/cm^2^ (51)	LIFU
Mechanical index (MI)	Peak negative pressure at focus divided by the square root of the fundamental frequency	NA	1.9 (51)	LIFU
Thermal index	Acoustic power is divided by the acoustic power required to achieve a 1°C temperature increase for a given tissue	NA	6 (51)	LIFU
Spatial-peak pulse-average intensity (I_SPPA_)	Average intensity of the FUS waveform over pulse duration	NA	190 W/cm^2^ (51)	LIFU
Fundamental frequency	Frequency of the ultrasound transducer	Frequency, FUS frequency, carrier frequency, transducer frequency	NA	Both
Pulse repetition frequency (PRF)	Number of FUS pulses that occur within 1 s of stimulation	Reciprocal of the pulse repetition interval (PRI)	NA	LIFU
Pulse duration (PD)	The time of a single pulse, generally in milliseconds	Pulse width	NA	LIFU
Duty cycle (DC)	Percentage showing how often during sonication duration the FUS signal is on	Can calculate using PD * PRF * 100%	NA	LIFU
Temperature at focus	The temperature at the focus is due to HIFU stimulation	Focal temperature, max temperature	NA	HIFU
Thermal dose	Temperature at focus over a defined period of time	NA	NA	HIFU
Session duration	How long the subject is stimulated with the given ultrasound parameters	Sonication duration, stimulation duration, experimental time	NA	Both

FDA limits are included for the safety parameters. Commonly used terms for the same parameter are displayed. Additionally, the usage of each parameter for the different therapeutic types of FUS is displayed.

In addition to safety (maximum intensity output) for therapeutic ultrasound, some other FUS parameters, mostly for LIFU, are a current focus of investigation for optimizing the neural effect desired (e.g., inhibitory neuronal response, excitatory neuronal response, transient response, permanent) at the target while minimizing the size of the ultrasound focus (50, 52, 55–57). The FUS field has various terms for some of the same parameters and there are no strict criteria for reporting parameters used in a study (50). Therefore, it is important to understand each stimulation parameter and the effect different stimulation parameters have had in the varying brain regions when designing protocols to achieve the desired purpose. A summary of the safety parameters, commonly used stimulation parameters, common terms for parameters, if applicable, the FDA limits, and the relevance to the type of therapeutic FUS is shown in Table 1 (50, 51).

When designing pulsing protocols for the FDA-approved application of HIFU, the most important parameter is the peak temperature to create a lesion in the tissue, which occurs around 55–60°C (22). Peak temperature cannot directly be controlled, but both sonication duration and power independent of each other have shown to increase peak temperature by increasing (58, 59). The size of the lesion is another important characteristic, which is routinely evaluated using magnetic resonance thermal imaging throughout and following the procedure, with the desired size of most lesions being in the range of a few millimeters (mm) (22). Additionally, the sonication duration can be adjusted to affect the lesion (22). FUS parameters, besides safety, play an important role in governing the desired outcome.

For LIFU pulsing protocols, most procedures are currently performed at low pressures (less than 0.6 MPa) at the ultrasound focus. However, some studies have used stimulation protocols above 1 MPa for seizure suppression (37). LIFU pulse durations are usually less than or equal to 300 milliseconds (53). The increase in temperature with these parameters is small - only an increase of less than or equal to 0.01°C has been recorded (53). LIFU generally has a pulsing protocol with longer durations during the stimulation session than HIFU, on the scale of minutes over seconds (53). Generally, an increase in the time of LIFU stimulation increases the behavior response seen (53). Individual pulse length and the stimulation duration for a session are important considerations when determining a pulsing protocol for the desired outcome.

Fundamental frequency (transducer frequency) can determine the spatial length of the ultrasound focus and can impact the effectiveness of the FUS stimulation (53). Generally, the fundamental frequency is below the MHz range (53). For pulsing protocols and focal areas of several millimeters in humans, a typical frequency is between 250 and 500 kHz for LIFU (53). Having a shorter wavelength creates a sharper spatial focus with a higher frequency (53). Lower frequencies tend to penetrate through the skull more effectively than higher frequencies. FUS parameters are important in designing studies, ensuring the desired outcome, and translating findings to the clinic.

## 5 Limitations of focused ultrasound for the treatment of epilepsy

FUS holds tremendous promise for DRE. However, a few major limitations noted above and discussed here, mostly for LIFU, need to be considered before executing a FUS protocol. The benefit of FUS, both HIFU, and LIFU, is that the depth penetration allows the device to stimulate subcortical structures and has a small spatial resolution (~3 mm) when compared to other noninvasive devices, such as transcranial magnetic stimulation (52, 60, 61). However, the skull creates a barrier for the ultrasonic waves (60, 61). The impedance from the skull causes ultrasonic wave attenuation, refraction, and dispersion, which creates an unknown delivered dose of intensity at the focus (61). Investigators in the field of FUS are actively working to correct for the attenuation of intensity created by the skull through computational methods that correct for the intended dose at the target tissue and develop new devices that compensate for different skull thicknesses between subjects (62, 63).

LIFU has been shown to modulate neural activity; however, it has also been shown to stimulate the auditory network (40, 64, 65). Activating the cochlear pathway of the auditory network by vibrations across the skull that occur during LIFU stimulation is called an “auditory artifact” (64–67). Several approaches have been used to control for this artifact. The auditory artifact can be corrected following transection of the auditory nerves or removal of the cochlear fluid, utilizing an envelope for the ultrasonic waveform pulse regime to minimize abrupt pulsing transition, and/or stimulation at an off-target brain area (40, 64, 66–68). Besides work by Murphy et al. (40), groups that have researched FUS application in evoked seizure or epilepsy preclinical models have either not or inadequately controlled for this artifact (28–30, 32, 34, 36, 38, 40, 69). Thus, there is a need for careful interpretation of the findings and properly controlled experiments before concluding that targeted LIFU is sufficient to modulate seizure activity.

## 6 Future directions for FUS therapy for DRE

While HIFU is FDA-approved to treat movement disorders, preclinical models of epilepsy could provide a new avenue of study for MRgFUS to understand treatment for DRE (70, 71). Animal studies provide the means to study phenotypes and syndromes that we cannot study in humans (70, 71). By using preclinical animal models, the field could determine the preferred brain targets for this line of therapy, the optimal FUS parameters (i.e., peak temperature, temperature rise, thermal dose, etc.) for correct lesion sizes, and long-term side effects of treating DRE with HIFU before clinical translation. Determining the effectiveness of HIFU for DRE could decrease barriers to surgery and side effects with deeper and smaller targets, such as the fornix, for ablative surgery with HIFU (3, 11, 13–15, 26).

Optimization of LIFU parameters to achieve the desired outcomes in epilepsy is still needed. LIFU has been shown to inhibit and excite brain circuits with different stimulation parameters across different brain areas and networks (i.e., excitation of the motor cortex, decreased seizures) (28–32, 34–37, 57, 72–74). LIFU has also been shown to act on various mechanosensitive and voltage-gated ion channels (56, 75–79). At the cellular level, there has been minimal work done to show the effects of LIFU stimulation on various neuronal cell types, and it has been suggested that LIFU can activate cell types beyond neurons (e.g., astrocytes) (80). These neuronal supporting cells are investigated in the search for new therapeutic targets for epilepsy (81). Therefore, optimizing FUS parameters to stimulate and alter the function of these cells with LIFU could be an interesting direction of study (80–82). Murphy et al. (40), created a device that allows imaging to be performed during LIFU stimulation, and a proof of concept was performed in the intrahippocampal kainate mouse model of epilepsy, showing brief suppression of neural activity in the hippocampus (40). Techniques such as coupling imaging with LIFU would provide the opportunity to research the mechanisms of LIFU stimulation in the epilepsy network (40). Understanding how specific LIFU parameters disrupt the epileptic network at the neuronal activity, cellular, and molecular levels may inform us of the appropriate stimulation paradigms for the ultimate treatment of epilepsy.

Ablation of epileptic foci with HIFU could potentially be a direct substitute for invasive or minimally invasive resection surgeries in people with DRE. Additionally, targeted therapy with LIFU could provide novel treatments for people with DRE, such as targeted-drug delivery to seizure-generating brain regions (43). These therapies are combinational therapies (stimulation + nanoparticle encapsulated drug or nonthermal lesioning) and may provide a localized effect rather than the systemic effects of current anti-seizure medications (ASMs) (43). Additionally, investigating the effects of ASMs when delivered locally to determine if there is a change in drug resistance, antiepileptic effects, and/or unwanted side effects may be of potential therapeutic benefit. Combinational therapy also opens the door for new experimental avenues. Targeted-drug delivery with nanoparticles can be used in brain mapping and could provide an important research tool for understanding the seizure-generating and/or comorbid neural networks (46). Combinational therapies show promise in numerous clinical and experimental applications.

The current future directions of HIFU and LIFU indicate the exciting potential applications for experimental and therapeutic techniques for DRE.

## 7 Conclusion

FUS is a completely noninvasive approach that can be used for both surgical and nonsurgical neuromodulation therapies using both thermal and nonthermal energy (19). Additionally, FUS can be used to reversibly and locally perturb the BBB to allow focused delivery of ASMs and investigational molecules to the seizure foci (43). While MRgFUS is the commonly used HIFU device and is FDA-approved for the surgical treatment of essential tremor and Parkinson’s disease, it is in the early days of clinical epilepsy research, with one Phase-1 open-label trial using MRgFUS targeting the anterior nucleus of the thalamus and one report derived from a retrospective study that used a theoretical modeling study to demonstrate the potential benefits of ablating the fornix/fimbria connection for DRE (27, 83). However, numerous LIFU studies in rodents and nonhuman primate studies have shown seizure suppression, and clinical trials for LIFU intervention for people with DRE are currently planned (28–32, 37, 40, 45). Details of clinical trials and preclinical and case report studies relating to FUS effects on seizures and epilepsy have been summarized in Supplementary Tables S1, S2, respectively. Future studies elucidating the cellular mechanisms through which LIFU modulates neuronal activity will also drive innovation and improve safety and efficacy. Thus, future work is poised to determine which FUS applications may be beneficial in treating DRE.

## Author contributions

CC: Conceptualization, Data curation, Funding acquisition, Writing – original draft, Writing – review & editing. EF: Data curation, Writing – review & editing. JR: Writing – review & editing. KW: Conceptualization, Writing – review & editing, Funding acquisition.
