# Supplementary material for: Ultrasonic therapies for seizures and drug-resistant epilepsy
Source: Front Neurol. 2023 Dec 12;14:1301956. doi: 10.3389/fneur.2023.1301956 (PMC10756913; doi:10.3389/fneur.2023.1301956)
Supplement: Supplementary file 3 [file Data_Sheet_1.docx]

Supplementary Material

Ultrasonic Therapies for Drug-Resistant Epilepsy

Carena Cornelssen^1, 2^, Eli Finlinson^1,2^, John D. Rolston^1,3^, Karen S. Wilcox^1,2,4*^

*** Correspondence:** Karen S. Wilcox: karen.wilcox@hsc.utah.edu

# Supplementary Tables

**Table S1**: Summary of HIFU and LIFU clinical trials for drug-resistant epilepsy (DRE). This table summarizes current trials sorted by FUS therapy type. Information on the trial includes clinical trial identifier number, patient condition as it relates to DRE, age of participants in the study, study time frame, brain target location, FUS therapy type, current recruitment status of the study, location of the study, and FUS device type. Items unable to be located were indicated with the abbreviation “NF” for not found.

**Table S2**: Summary of HFU, LIFU, and targeted-drug delivery mediated by FUS studies for DRE. This table summarizes current published work sorted by FUS therapy type. Information on the study includes the species used, model of epilepsy or seizure used, brain area targeted, ultrasound transducer used, FUS parameters used if available in the publication, and major findings from the publication. Abbreviations used in table S2 include, pentylenetetrazol (PTZ), kainic acid (KA), Mechanical Index (M.I.), Thermal Index (T.I.), Spatial-peak temporal-average Intensity (I_SPTA_), Spatial-peak pulsed-average Intensity (I_SPPA_), Fundamental Frequency (FF), Tone Burst Duration (TBD), Pulse Repetition Frequency (PRF), Not Found (NF).
